# Supplementary material for: Transcriptome Analysis Revealed the Early Heat Stress Response in the Brain of Chinese Tongue Sole (Cynoglossus semilaevis)
Source: Animals (Basel). 2023 Dec 26;14(1):84. doi: 10.3390/ani14010084 (PMC10777917; doi:10.3390/ani14010084)
Supplement: Supplementary file 1 [file animals-14-00084-s001.zip › File S1/KEGG enrichment analysis of time-series cluster 1, 2, 4 in both sexes.pdf]

In addition to KEGG analyses of the combined clusters, we also performed KEGG enrichment analyses of each of the eight clusters of male and female. (Figure 4A, B and Figure S3) show that the pathways enriched in female cluster 1 mainly include Spliceosome, DNA polymerase and Nucleotide excision repair. The pathways enriched in Cluster 2 mainly include Cell cycle, DNA replication, and Fat digestion, whereas Cluster 3 was mainly enriched for Cytokine-Cytokine receptor interaction, IL-17 signaling pathway, and ECM-receptor interaction. Cluster 4 demonstrated enrichment in pathways such as Neuroactive-ligand-receptor interaction, cAMP signaling pathway, and Signaling pathways regulating pluripotency of stem cells. Male cluster 1 exhibited enrichment mainly in pathways including Spliceosome, DNA polymerase, and DNA degradation. Cluster 2 displayed enrichment in Metabolic pathways, DNA replication, and Fatty acid metabolism. Cluster 3 exhibits enrichment for the Cytokine-Cytokine receptor interaction, Aldosterone synthesis and secretion, and PI3K-Akt signaling pathway. Additionally, it is mainly enriched for the Cytokine-Cytokine receptor interaction, Aldosterone synthesis and secretion, and PI3K-Akt signaling pathway, alongside the IL-17 signaling pathway. We can observe that even clusters displaying the same trend in the same sex do not exhibit enrichments in the same pathways. Examples include Cluster 1 and Cluster 2 in females and so on. Regarding the clusters themselves, pathways enriched in female cluster 1 and male cluster 1 are somewhat analogous, as well as in female cluster 2 and male cluster 2. This indicates that the pathways enriched in clusters of different sexes are similar in the overall downward trend. In contrast, female cluster 3 and male cluster 3 exhibit distinct enriched pathways, possibly attributed to varied peak time points. The divergence in enrichment pathways between female cluster 4 and male cluster 4 could be attributed to the dissimilar overall trends within the clusters.

We conducted KEGG enrichment analyses for eight clusters, including males and females. Heat stress is a physiological response to abrupt fluctuations in external temperature. Our objective was to identify genes or pathways in the brain that demonstrate quick response in the early stages as they have the potential to regulate downstream genes leading to phenotypic changes. Therefore, we narrowed down our focus to clusters with a rising overall trend. We aimed to investigate high-temperature early response genes in the brains of *C. semilaevis*. To achieve this, we compared clusters 3 and 4 in females and chose the former. We then compared clusters 3 and 4 in males and also selected cluster 3. Hence, we selected cluster 3 for subsequent analyses in both males and females. Of course, we also performed PPI analyses for other clusters and put the results in Figure S4.
