# Supplementary material for: Transcriptome Analysis Revealed the Early Heat Stress Response in the Brain of Chinese Tongue Sole (Cynoglossus semilaevis)
Source: Animals (Basel). 2023 Dec 26;14(1):84. doi: 10.3390/ani14010084 (PMC10777917; doi:10.3390/ani14010084)
Supplement: Supplementary file 1 [file animals-14-00084-s001.zip › 附图/Figure S2/S2.pdf]

A

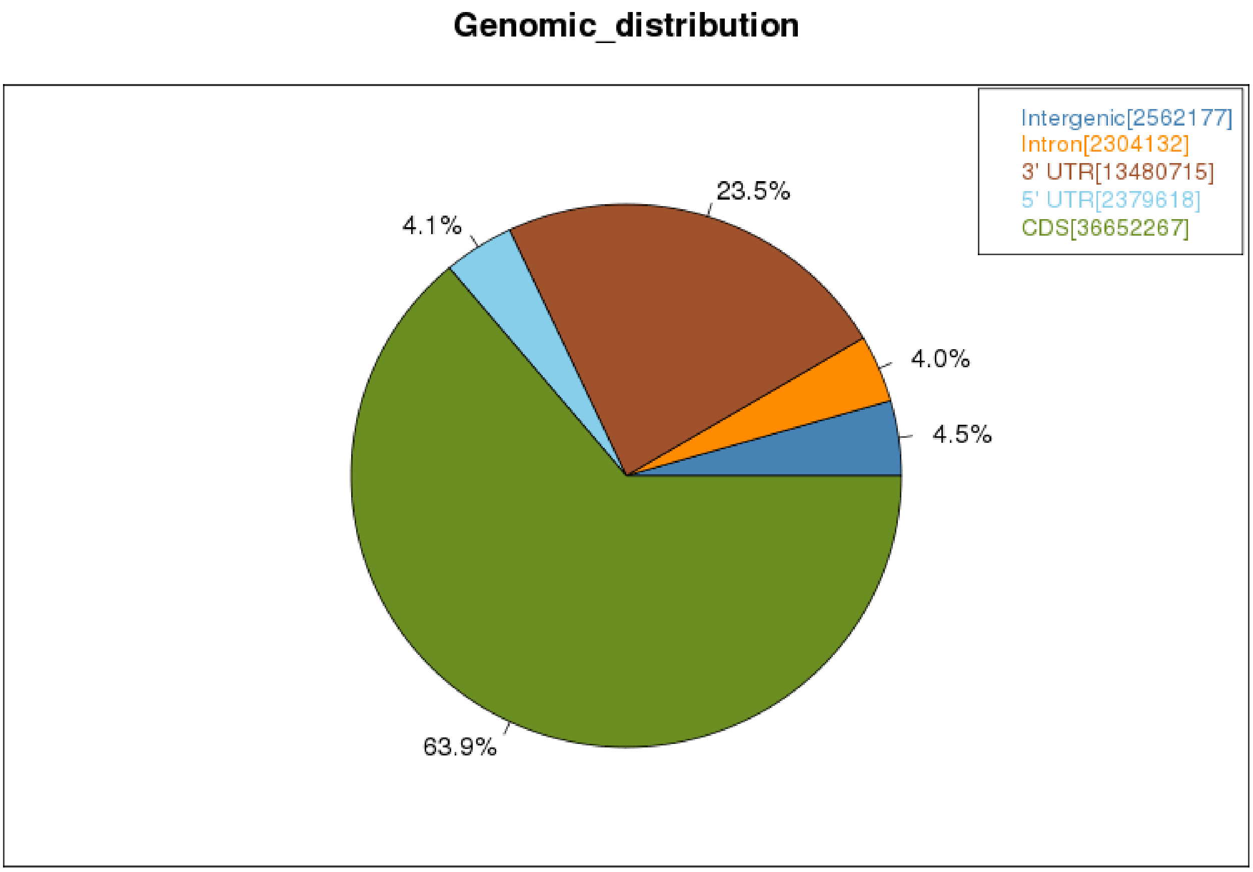

B

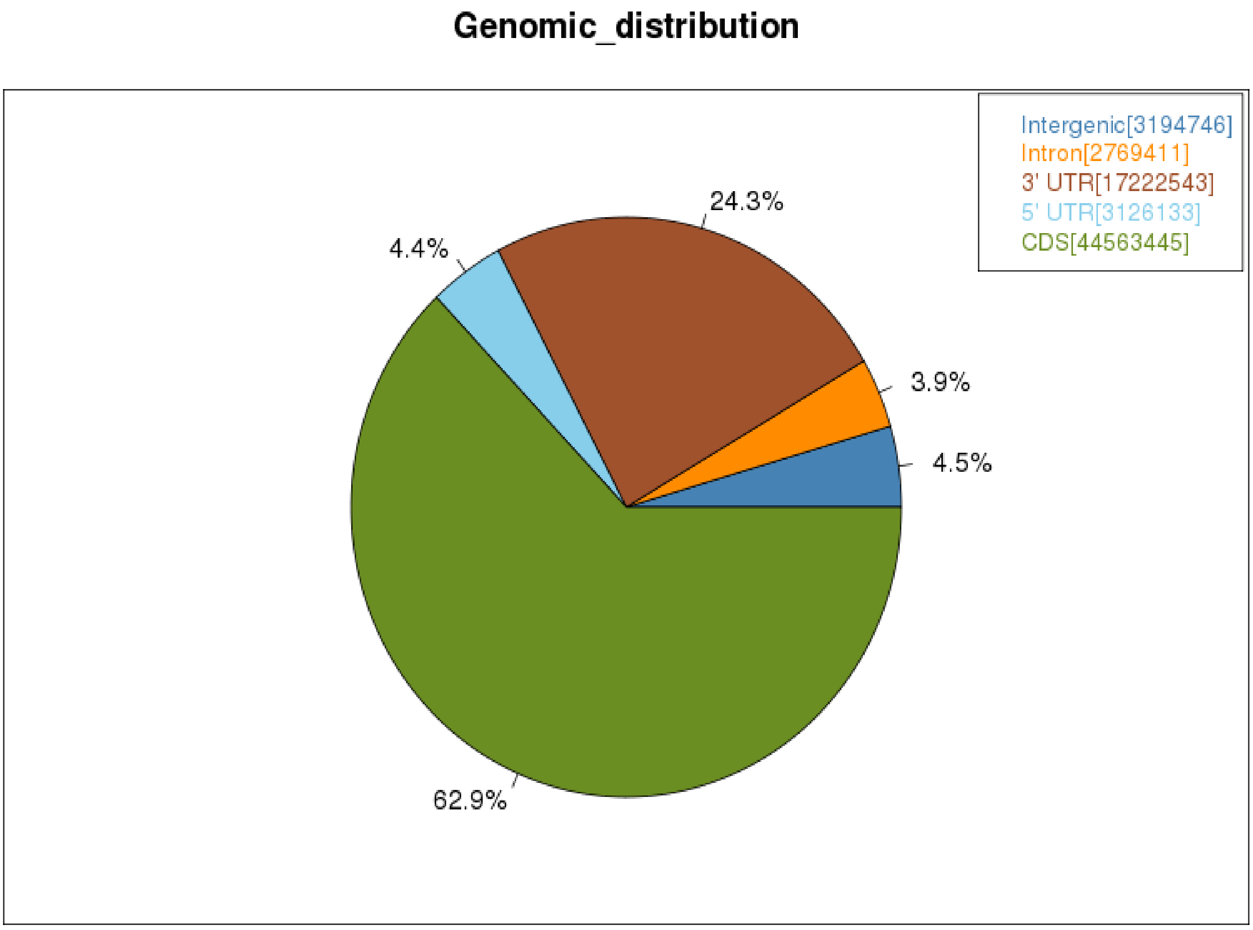

Figure S2. Genomic region distribution. A. Genomic region distribution in Female (Represented with HS1h-F2). B. Genomic region distribution in Male (Represented with HS2h- M1).
