# Supplementary material for: Transcriptome Analysis Revealed the Early Heat Stress Response in the Brain of Chinese Tongue Sole (Cynoglossus semilaevis)
Source: Animals (Basel). 2023 Dec 26;14(1):84. doi: 10.3390/ani14010084 (PMC10777917; doi:10.3390/ani14010084)
Supplement: Supplementary file 1 [file animals-14-00084-s001.zip › 附图/Figure S3/S3.pdf]

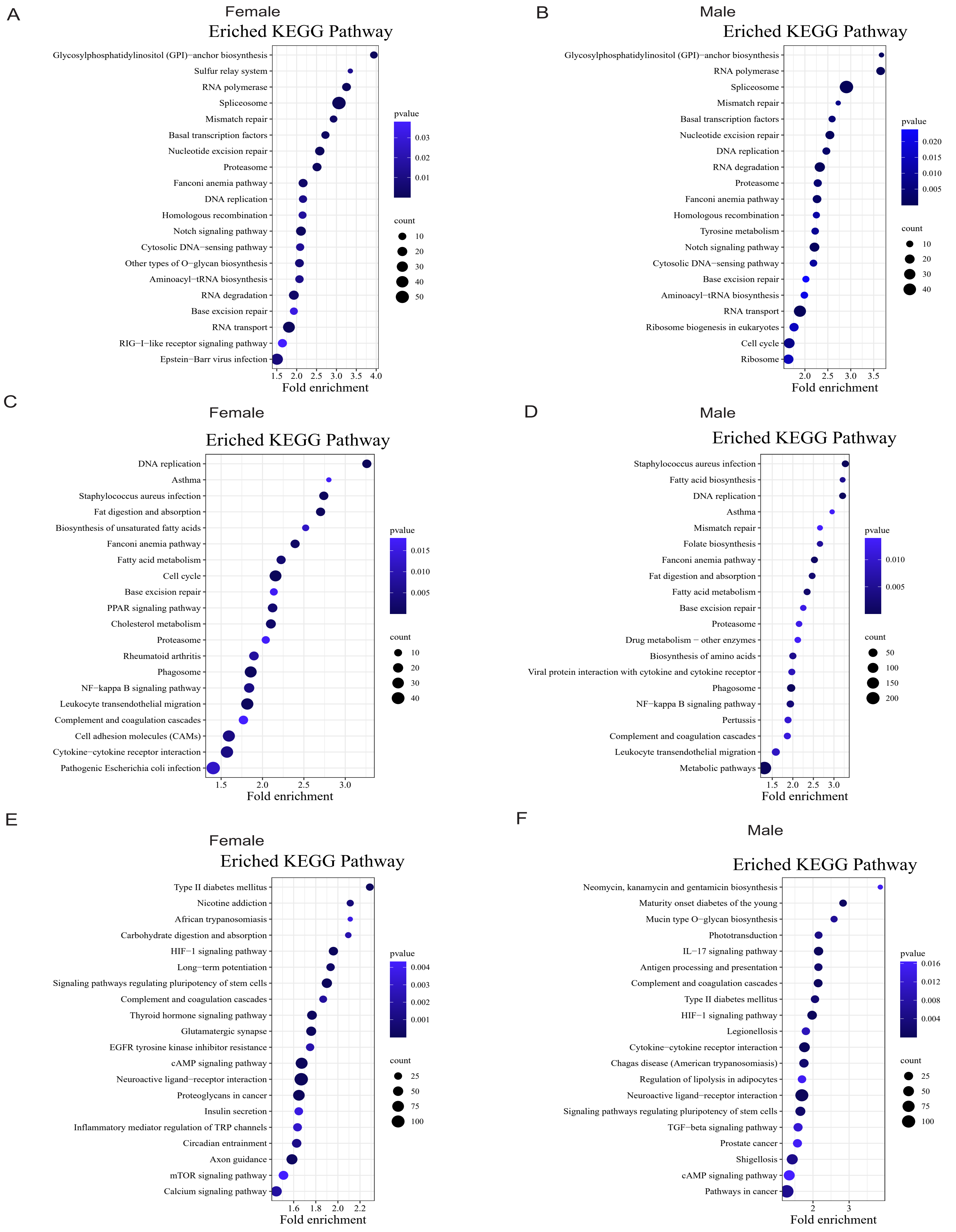


Figure S3. KEGG enrichment analysis in other clusters. A. KEGG enrichment analysis of DEGs contained in Cluster 1 of Female. B. KEGG enrichment analysis of DEGs contained in Cluster 1 of Male. C. KEGG e-nrichment analysis of DEGs contained in Cluster 2 of Female. D. KEGG enrichment analysis of DEGs con-tained in Cluster 2 of Male. E. KEGG enrichment analysis of DEGs contained in Cluster 4 of Female. F. KEGG enrichment analysis of DEGs contained in Cluster 4 of Male.
