# Supplementary material for: Transcriptome Analysis Revealed the Early Heat Stress Response in the Brain of Chinese Tongue Sole (Cynoglossus semilaevis)
Source: Animals (Basel). 2023 Dec 26;14(1):84. doi: 10.3390/ani14010084 (PMC10777917; doi:10.3390/ani14010084)
Supplement: Supplementary file 1 [file animals-14-00084-s001.zip › 附图/Figure S4/S4.pdf]

A

Female

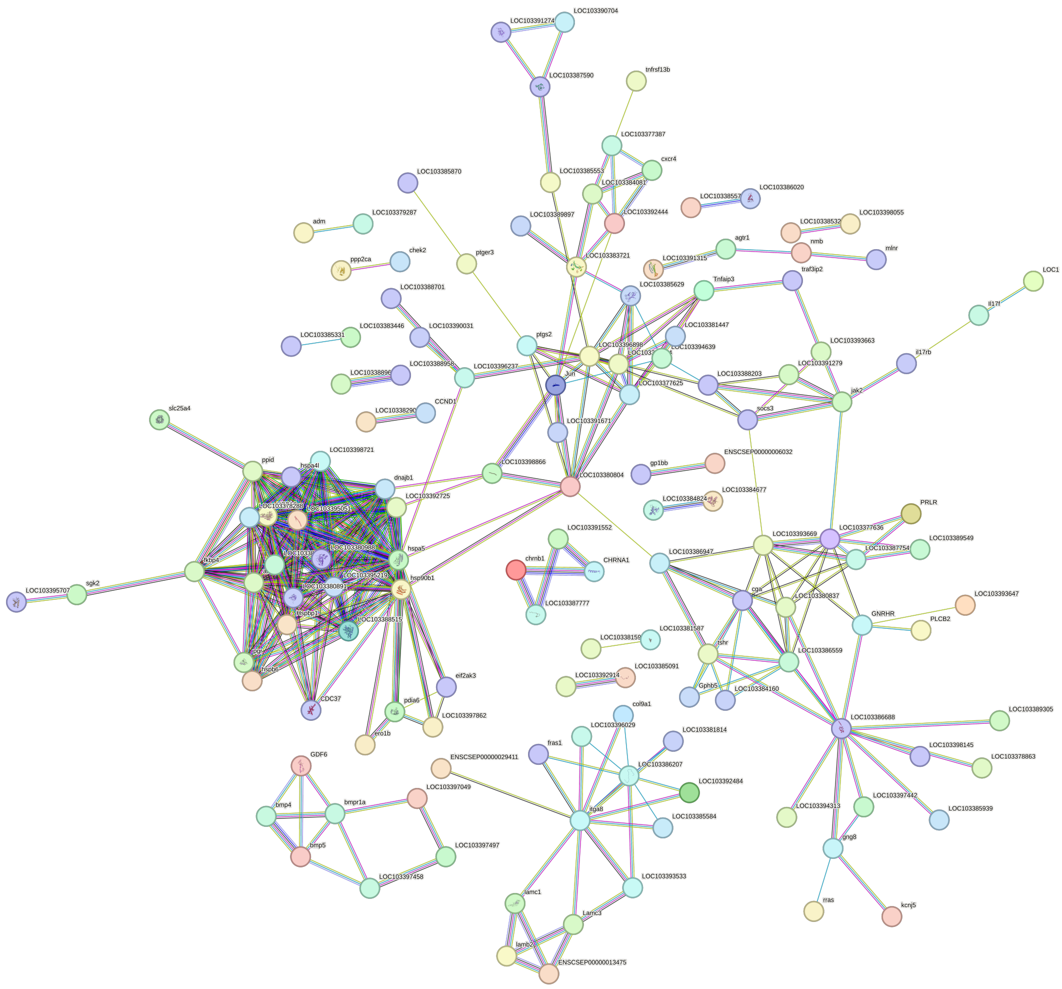

B

Male

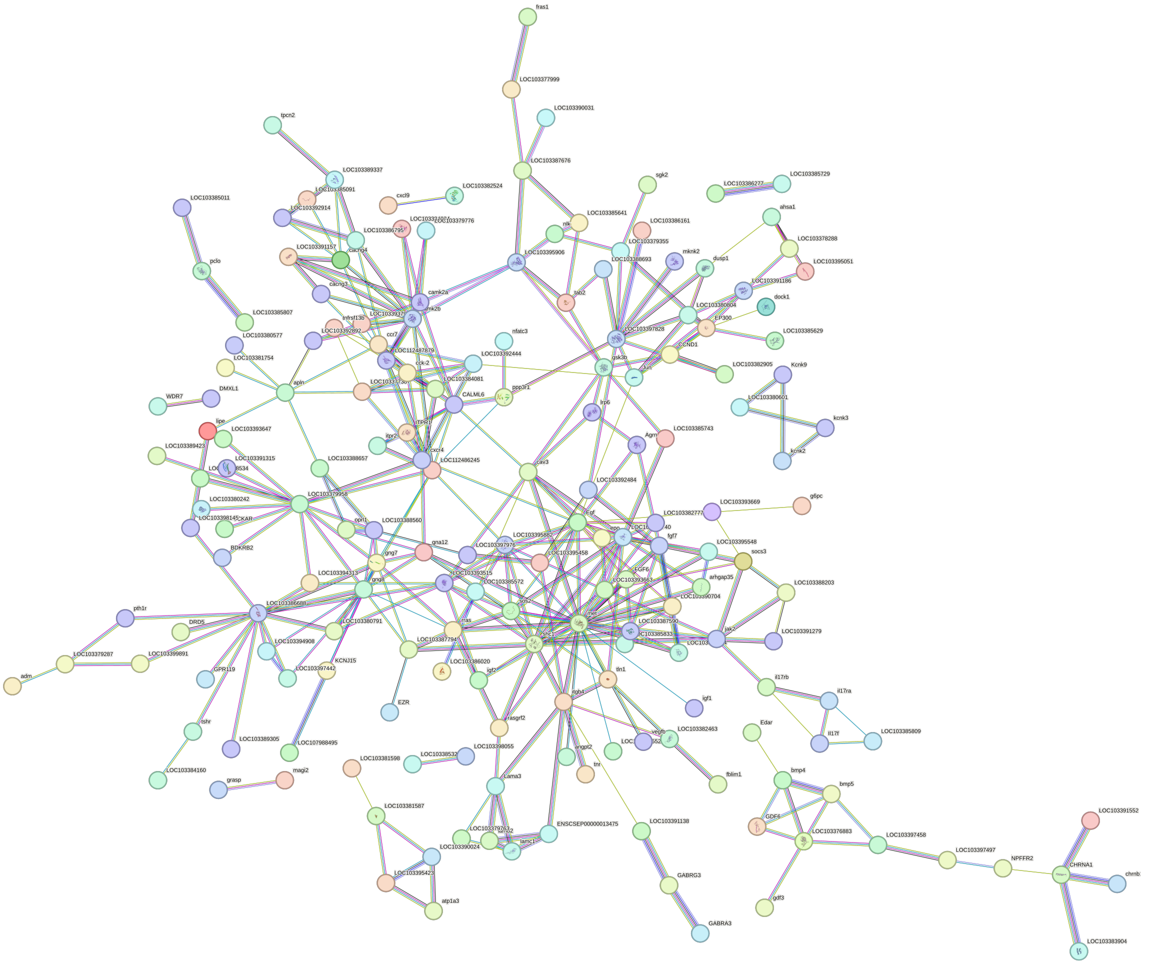

Figure S4. A. PPI networks constructed by Figure 4A. B. PPI networks constructed by Figure 4B.
