# Supplementary material for: Transcriptome Analysis Revealed the Early Heat Stress Response in the Brain of Chinese Tongue Sole (Cynoglossus semilaevis)
Source: Animals (Basel). 2023 Dec 26;14(1):84. doi: 10.3390/ani14010084 (PMC10777917; doi:10.3390/ani14010084)
Supplement: Supplementary file 1 [file animals-14-00084-s001.zip › 附图/Figure S5/S5.pdf]

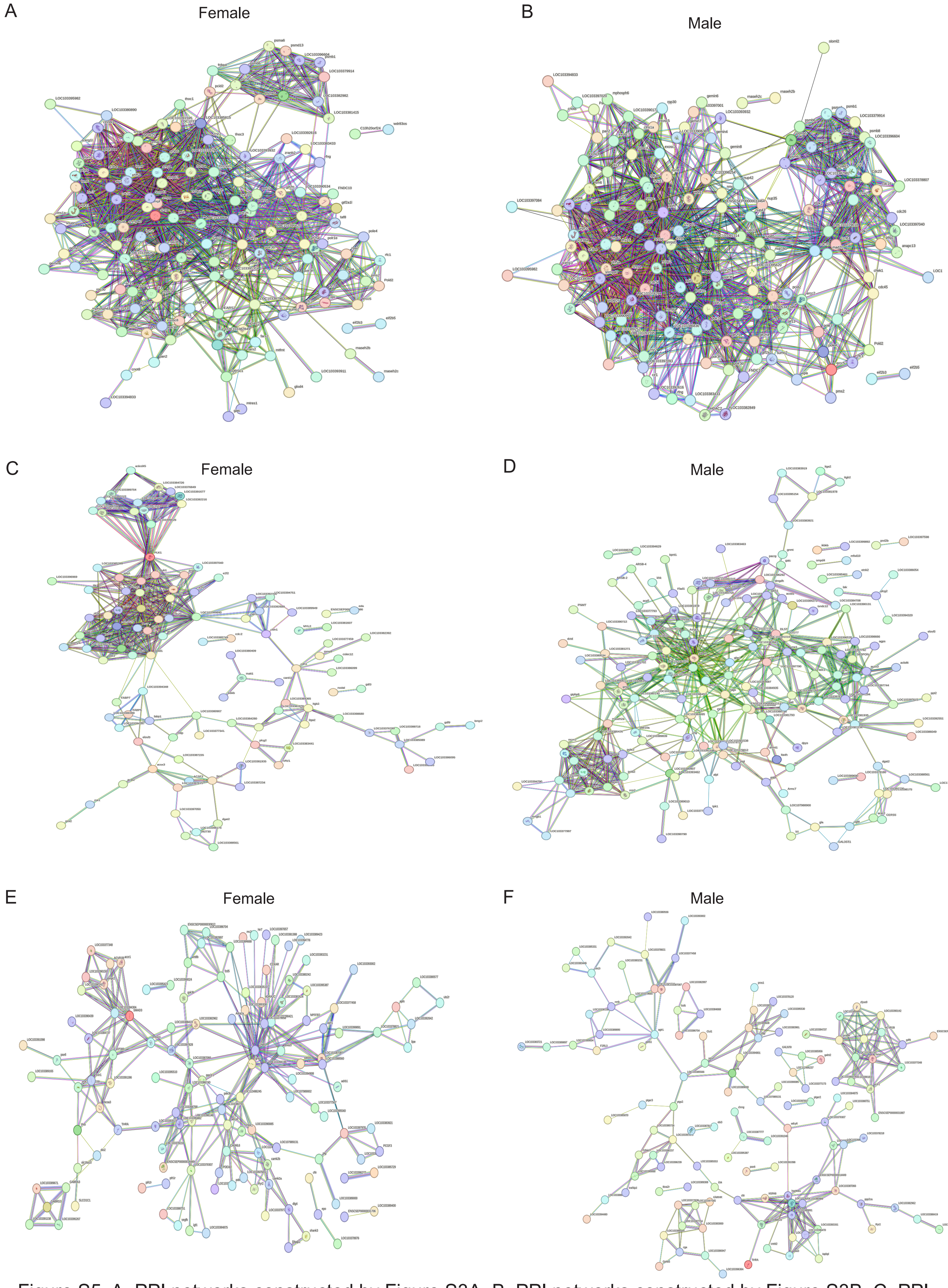

Figure S5. A. PPI networks constructed by Figure S3A. B. PPI networks constructed by Figure S3B. C. PPI networks constructed by Figure S3C. D. PPI networks constructed by Figure S3D. E. PPI networks constructed by Figure S3E. F. PPI networks constructed by Figure S3F.
