# Supplementary figures and images for: Transcriptome Analysis Revealed the Early Heat Stress Response in the Brain of Chinese Tongue Sole (Cynoglossus semilaevis)
Source: Animals (Basel). 2023 Dec 26;14(1):84. doi: 10.3390/ani14010084 (PMC10777917; doi:10.3390/ani14010084)

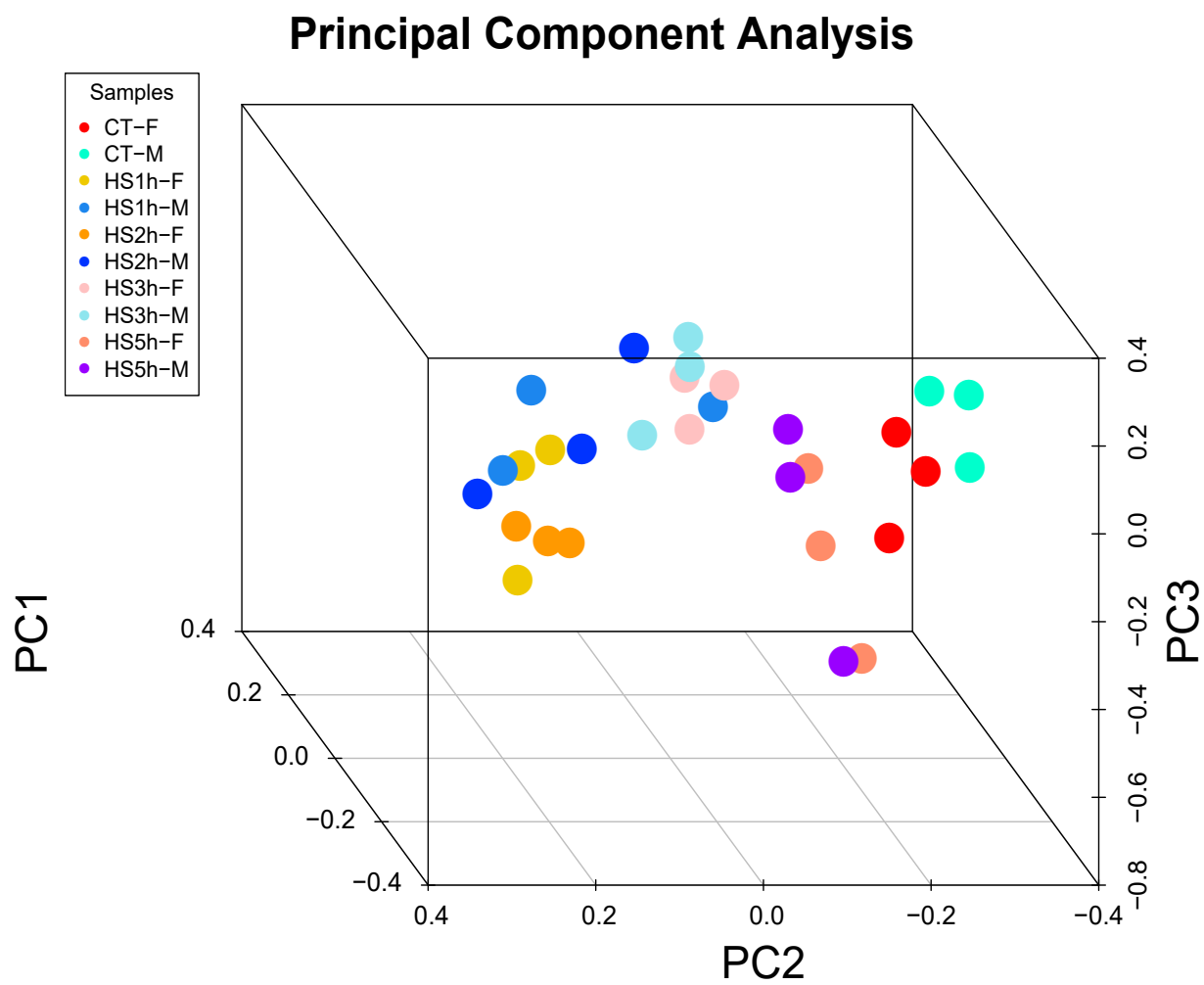

Figure S1. PCA analysis.

Supplement: Supplementary file 1 [file animals-14-00084-s001.zip › 附图/Figure S1/S1.pdf]

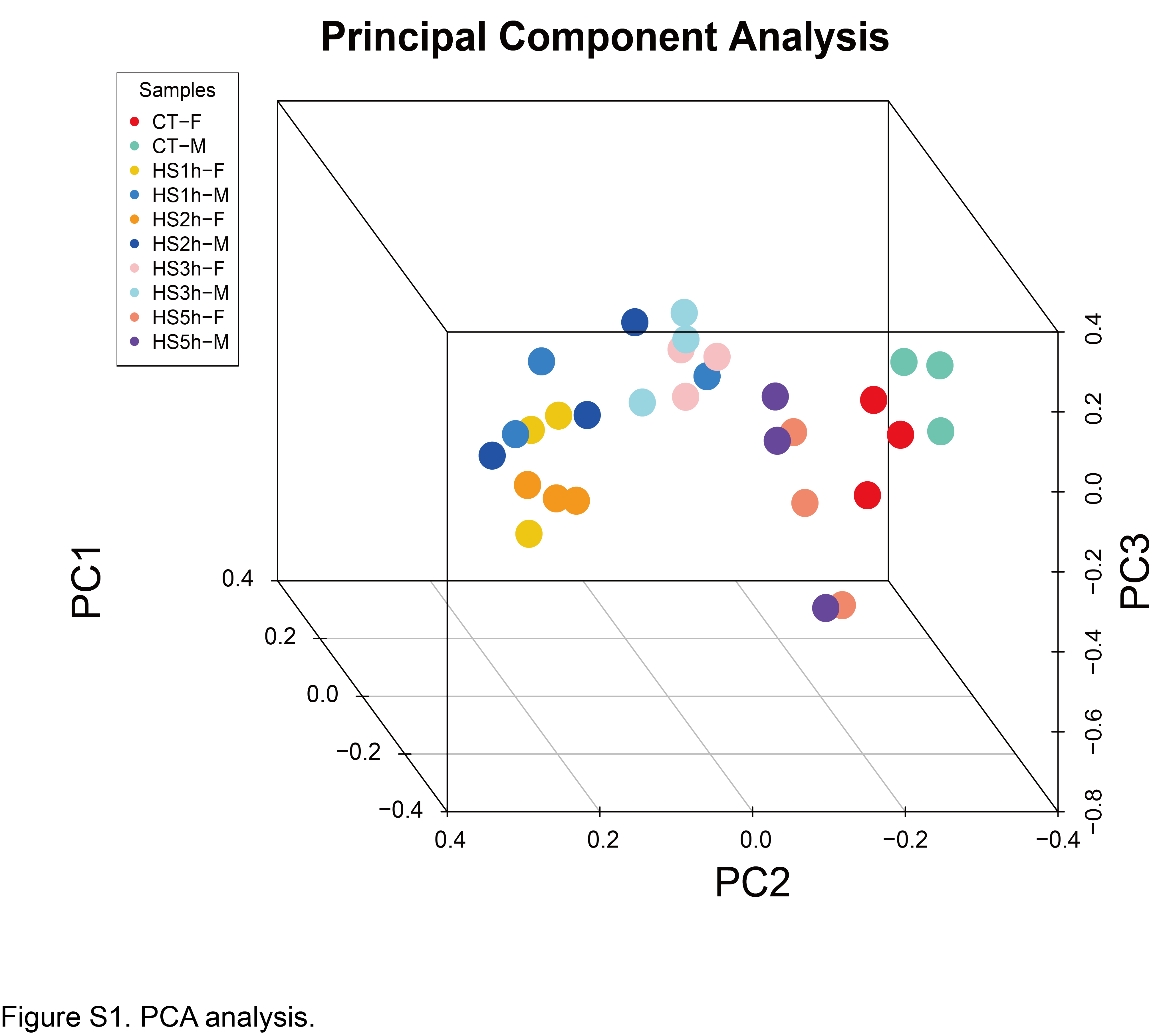

Supplement: Supplementary file 1 [file animals-14-00084-s001.zip › 附图/Figure S1/S1.png]

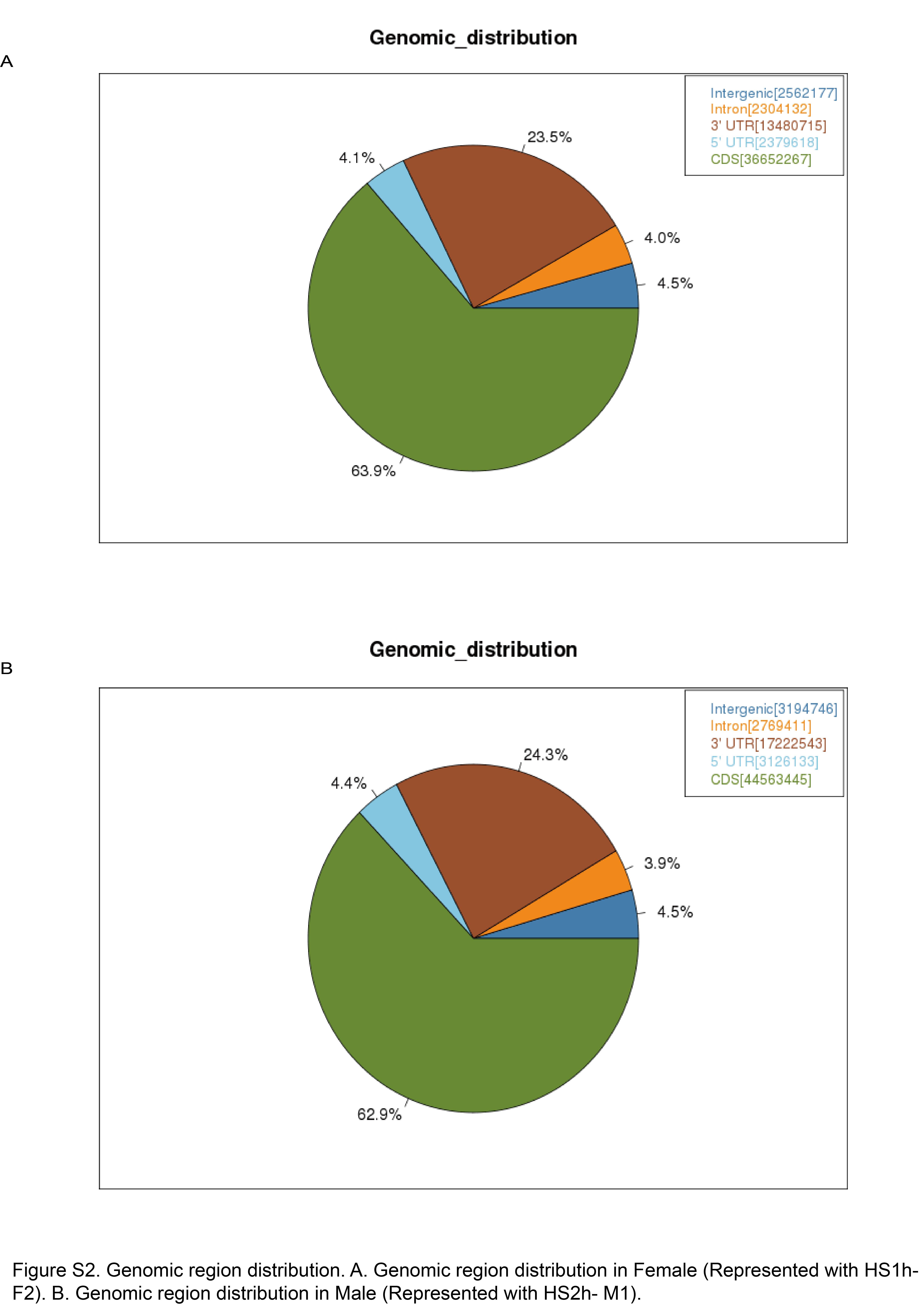

Supplement: Supplementary file 1 [file animals-14-00084-s001.zip › 附图/Figure S2/S2.png]

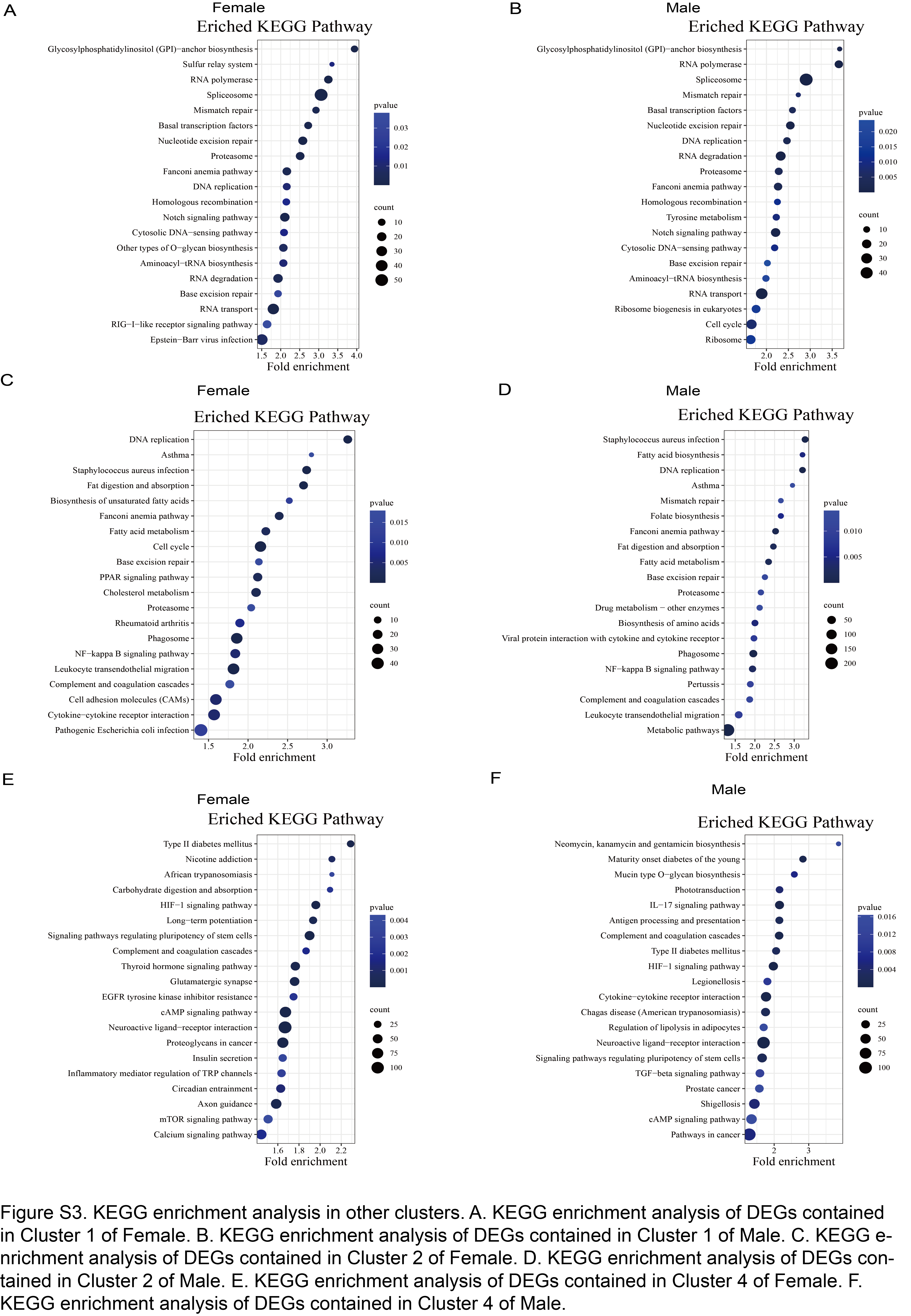

Supplement: Supplementary file 1 [file animals-14-00084-s001.zip › 附图/Figure S3/S3.png]

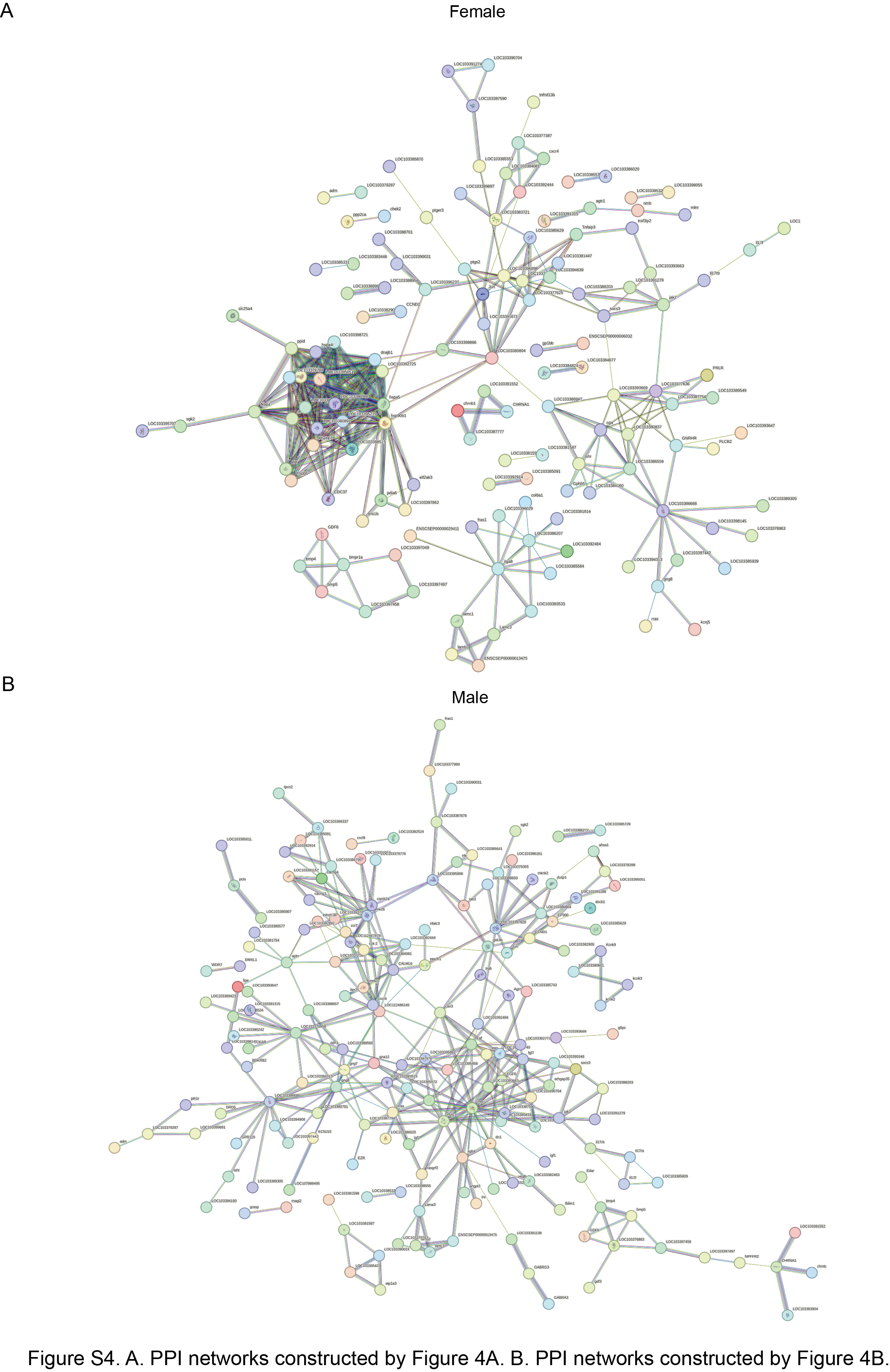

Supplement: Supplementary file 1 [file animals-14-00084-s001.zip › 附图/Figure S4/S4.png]

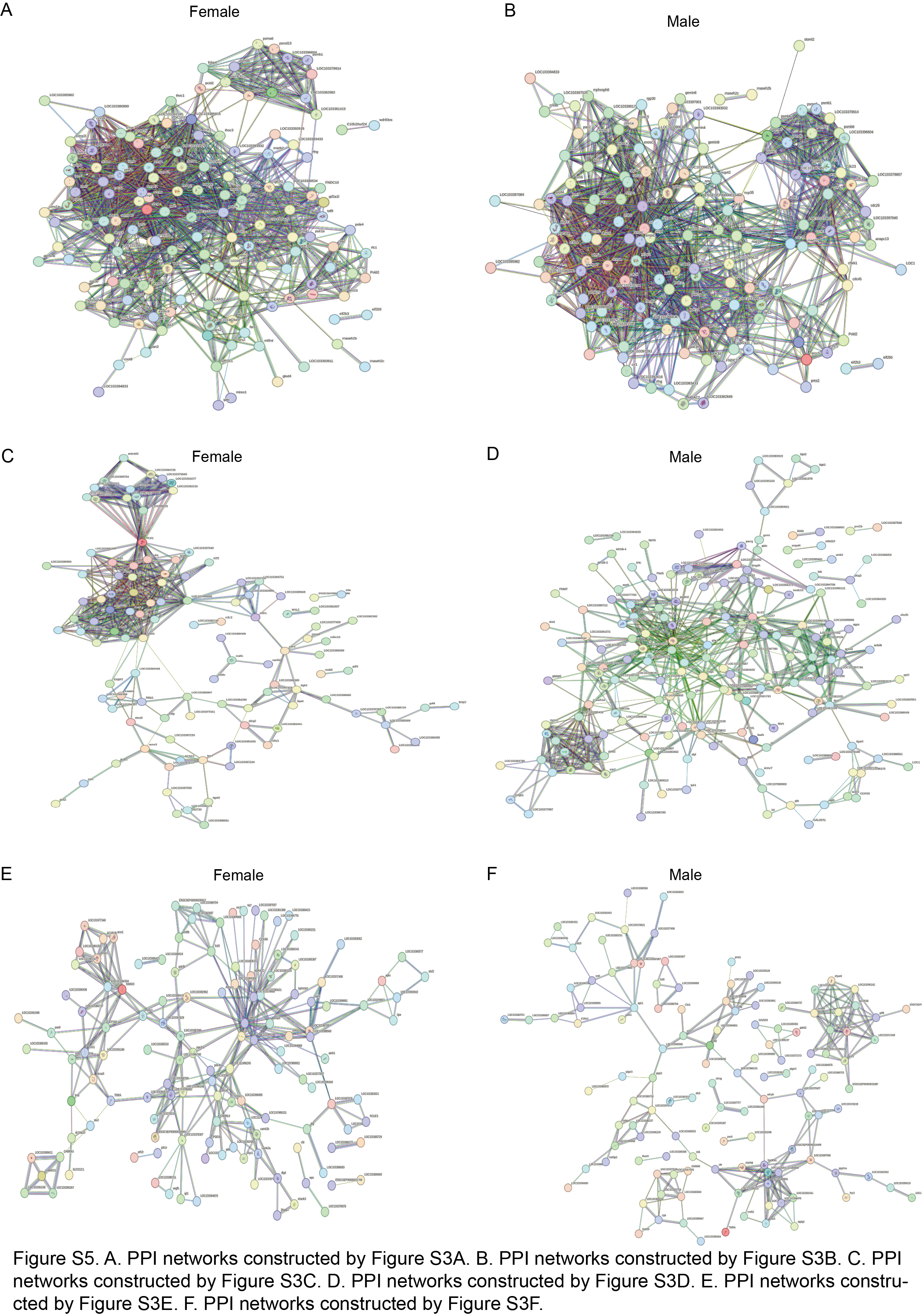

Supplement: Supplementary file 1 [file animals-14-00084-s001.zip › 附图/Figure S5/S5.png]

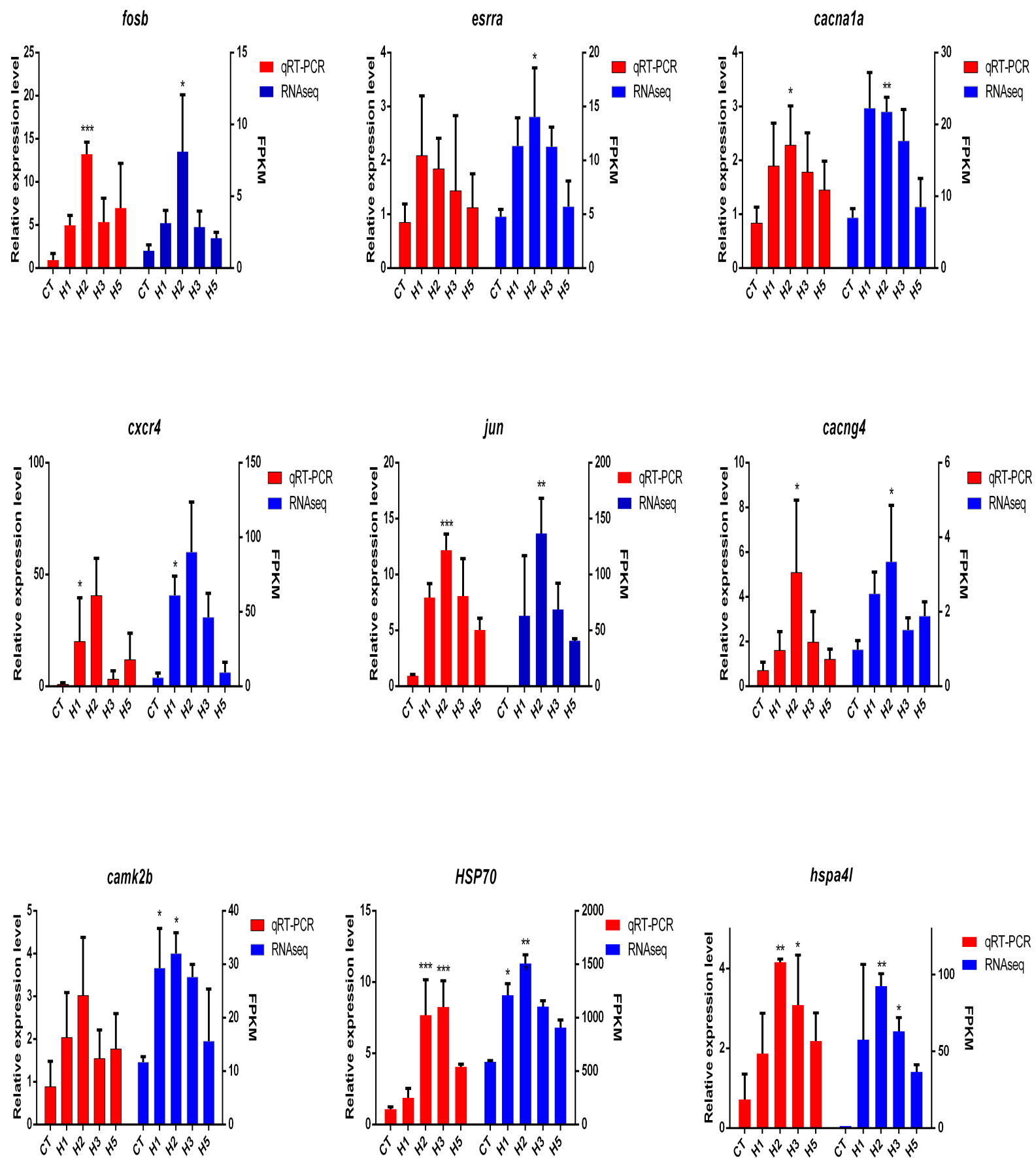

Figure S6. Real-time PCR verifications. (qRT-PCR), the expression patterns of nine DEGs.

Supplement: Supplementary file 1 [file animals-14-00084-s001.zip › 附图/Figure S6/S6.pdf]

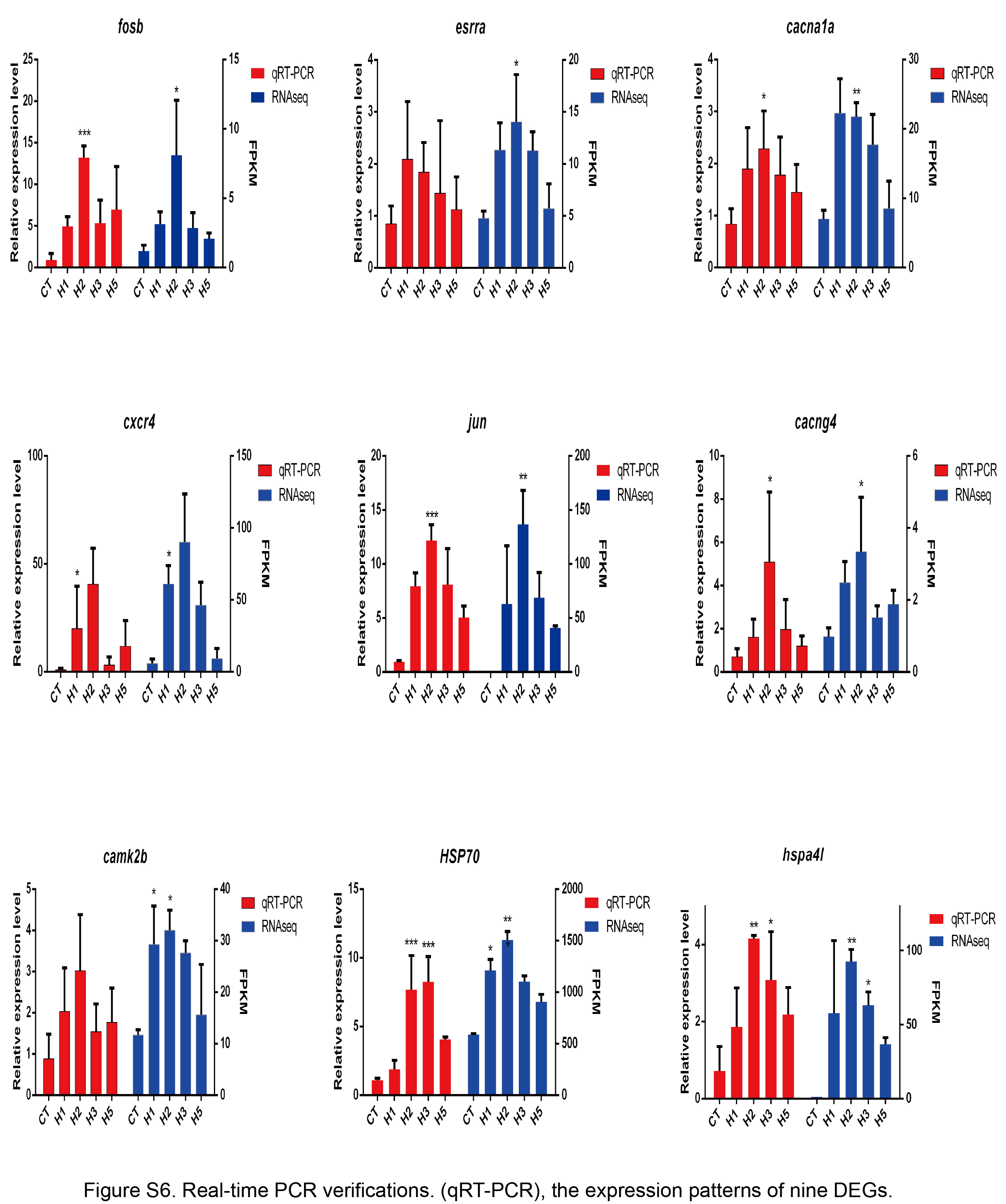

Supplement: Supplementary file 1 [file animals-14-00084-s001.zip › 附图/Figure S6/S6.png]
